# A novel minimally invasive fixation method for flail chest management in a Canine model: an animal research

**DOI:** 10.1186/s13019-023-02445-5

**Published:** 2023-12-14

**Authors:** Zhe Li, Weiwei Zhu, Bing Zhang, Yaxiao Zhang, Huixian Li, Baolei Lv, Qiang Zhen, Lin Liu, Lijun Liu, Yanxin Wu, Shujun Li

**Affiliations:** 1 Department of Thoracic Surgery, The Second Hospital of Hebei Medical University, No. 215 Heping West Road, Shijiazhuang City, Hebei 050000 China; 2 Department of Thoracic Surgery, Shijiazhuang People’s Hospital, No. 365 Jianhua South Street, Shijiazhuang City, Hebei 050031 China; 3 Department of Thoracic Surgery, The First Hospital of Shijiazhuang, No. 36, Fanxi Road, Shijiazhuang City, Hebei 050011 China; 4 Hebei General Hospital, No.348 Heping West Road, Shijiazhuang City, Hebei 050051 China; 5 Department of Thoracic Surgery, Hebei General Hospital, No.348 Heping West Road, Shijiazhuang City, Hebei 050051 China

**Keywords:** Multiple Rib fracture, Flail Chest, Surgical fixation, Interior stabilization, Intercostal muscle, Minimally invasive

## Abstract

**Background:**

Multiple rib fractures can lead to flail chest with up to 35% mortality rate due to severe pulmonary complications. Current treatments of flail chest remain controversial. Studies have shown that surgical treatments can improve outcomes and reduce mortality, comparing to non-operative treatments. Current surgical fixation methods focus on stabilization of ribs on the outward facing side, and they require division of intercostal muscles. Damages to surrounding nerves and vessels may lead to chronic pain. This study tests a novel interior fixation method that minimizes neurovascular injuries.

**Methods:**

Twelve healthy canines were divided in two surgical operation groups for exterior and interior fixation using titanium metal plates. Osteotomy with oblique fractures was prepared under general anesthesia. Exterior fixation was performed in open surgery. Interior fixation was minimally invasive using custom made tools including a flexible shaft extension screwdriver, solid plate stand, guiding wire loop and metal plates with threaded holes.

**Results:**

Respiratory and cardiovascular functions (RR, PO_2_, PCO_2_, SpO_2_, and HR) together with body temperature were measured before anesthesia and within 48 h after surgery. The difference in measurements was not statistically significant between the two groups before surgery with *P* values greater than 0.05. However, the interior group canines had better RR and PO_2_ values starting from the 24th hour, and better PCO_2_, SpO_2_, and HR values starting from the 48^th^ hour. It took longer operation time to complete the minimally invasive interior fixation surgery (*P* value less than 0.001), but the total blood loss was less than the exterior fixation group (*P* value less than 0.001). Results also showed that interior group canines suffered less pain, and they had quicker recovery in gastrointestinal and physical mobility.

**Conclusions:**

The investigative interior fixation method was safe and effective in rib stabilization on a canine rib fracture model, comparing to the exterior fixation method. The interior fixation was minimally invasive, with less damages to tissues and nerves surrounding the ribs, leading to better postoperative outcomes.

**Supplementary Information:**

The online version contains supplementary material available at 10.1186/s13019-023-02445-5.

## Highlights

Current metal prosthesis surgical fixation requires division of intercostal muscles, which may lead to neurovascular injuries and chronic pain.

Metal plate stabilization from the inward facing side of the rib avoids muscle division, and only needs a cut through a thin layer of periosteum to expose the bones.

A flexible soft shaft extension screwdriver can be bent, twisted, rotated, and turned into narrow space and dead end. It can be used with VATS in surgical rib fixation.

**Supplementary Information:**

The online version contains supplementary material available at 10.1186/s13019-023-02445-5.

## Background

Thoracic trauma accounts for 20-25% of trauma-related mortality [1]. Multiple rib fractures are common in thoracic injury, which can lead to Flail Chest with up to 35% mortality rate, due to severe pulmonary complications and sepsis [2, 3]. Flail Chest happens when 3 or more consecutive segmental rib fractures take place in more than one location, [4] and it is normally associated with paradoxical movement of the chest wall, reliance on mechanical ventilation, and prolonged ICU stay [5].

Current treatments of flail chest remain controversial. Supportive or non-operative treatments have been the predominant management options, which mostly involve pain control with analgesia, mechanical ventilation (both invasive and non-invasive), strapping, and packing [6–8]. Although being widely practiced, non-operative treatments can lead to higher rate of pneumonia, prolonged mechanical ventilation, ICU stay, and Acute Respiratory Distress Syndrome (ARDS) [9] resulting in severe morbidity and mortality [6, 8, 10].

Surgical or operative treatments have gained popularity in recent years, and studies have shown that early surgical intervention can significantly improve outcomes and reduce mortality [11, 12]. Surgical rib fracture fixation can be performed by thoracic surgeons or by a collaboration of multiple disciplinary, including trauma and orthopedic surgeons [13]. The advantages of surgical stabilization of rib fractures include reduced duration of mechanical ventilation, prevention of pulmonary complications, and the reduction of long-term pain, disability, or chest wall deformity.

There are a variety of surgical fixation methods available, but there’s not an optimal treatment option established yet [10, 14]. Absorbable fixation plates are bio-compatible, but they are sometimes not strong enough to hold displaced ribs in alignment [15]. A number of metal prostheses have been tested and clinically applied, including less rigid Kirschner wires, [16] and rigid metal hardware such as Judget Struts, [17] Intramedullary pins, [18] and a wide range of metal plates. The deficiencies of metal fixation include hardware being palpable in CT or MRI scans, stress shielding of plates, [19] and the potential need for removal in a follow-up operation [20, 21].

Most of the metal plates require division of intercostal muscles on the outward facing side of the ribs, resulting in neurovascular injuries and chronic pain. A metal prosthesis stabilizing ribs from the inward facing side of the ribs can avoid splints of muscles, thus causing less damages to nerves and blood vessels that concentrate on the exterior side of ribs.

The current study is designed to test a minimally invasive method and novel surgical tools for the operative treatment of rib fracture. The effective stabilization of rib fractures can lead to successful management of flail chest. A novel VATS-assisted interior surgical fixation method is applied on a canine rib fracture model, in comparison to the exterior fixation method. Operation safety, efficiency and postoperative outcomes were compared between the two methods.

## Methods

The animal experiment was carried out in accordance with the revised Animals (Scientific Procedures) Act 1986 in the UK and Directive 2010/63/EU in Europe. With approvals from the Institutional Animal Care and Use Committee of Hebei Medical University, and from the Research Ethics Committee of the Second Hospital of Hebei Medical University, 12 healthy male canines were provided by the University. They were randomly divided into two groups with proper labeling: the Exterior Group (EG), in which the metal plates were applied on the outward facing side of the rib; the Interior Group (IG), in which the metal plates were applied on the inward facing side of the rib.

### Material

**Fig. 1:**
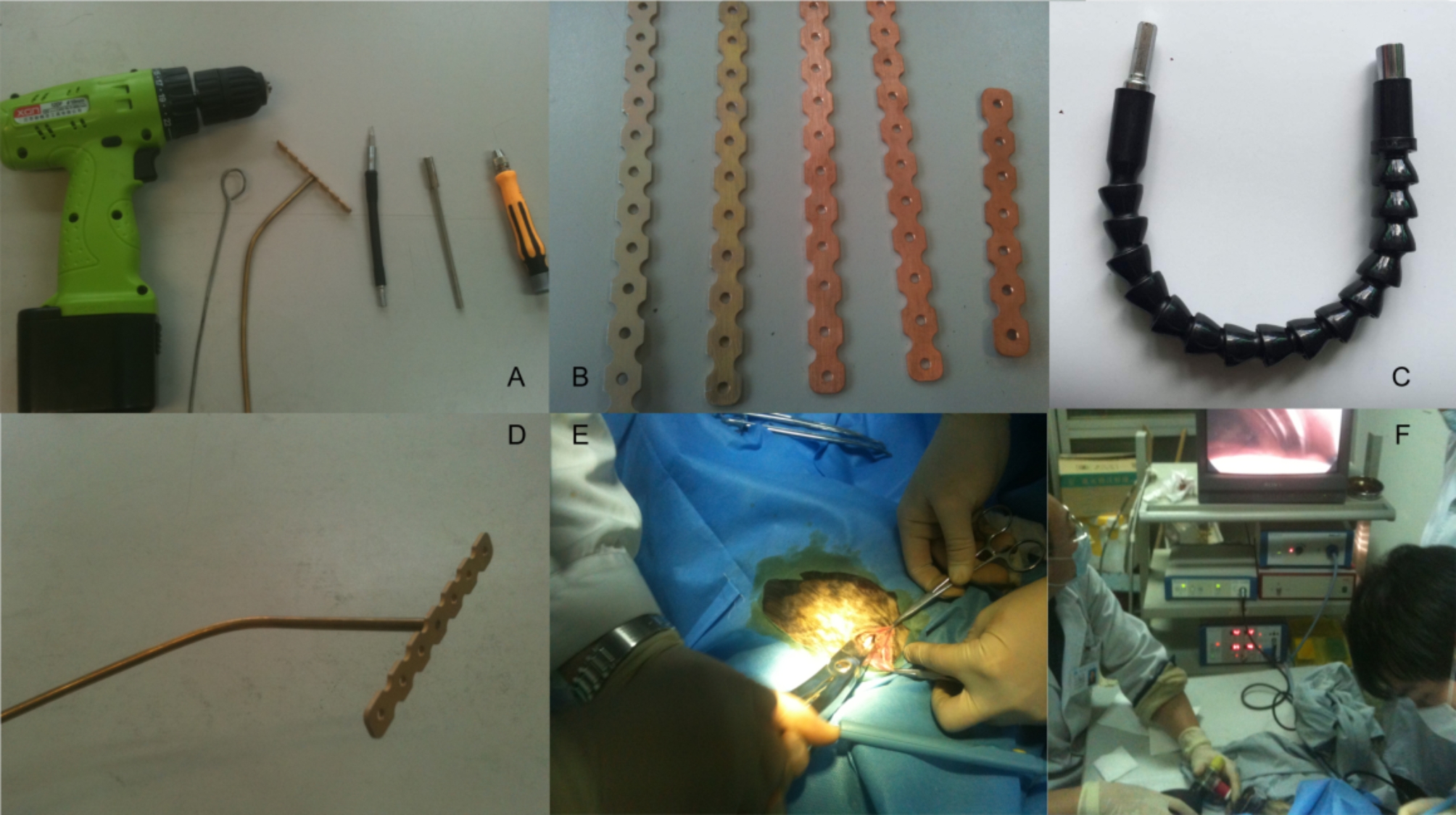
** A**: Tools used in operation; **B**: Metal plates of various sizes; **C**: Flexible soft shaft extension; **D**: Solid plate stand screwed onto threaded plate; **E**: Operation; **F**: VATS assisted operation

The medal plates used in the experiment were custom made with titanium to fit the size and shape of the canine rib. Custom made tools, devices and operation scenes were as shown in Fig. 1, including a cordless electric drill, guiding wire loop, threaded metal plates, flexible soft shaft extension, and solid plate stand screwed onto a threaded plate.

## Methods

### Preoperative preparation

#### Preparation before anesthesia

Food was withheld for 24 h, and water was withheld for 6 h before surgery in order to reduce the possibility of regurgitation and aspiration pneumonia. All canines were closely monitored for signs of anxiety, hypothermia (defined as body temperature below 37.2 °C), and dehydration [22].

#### Premedication before general anesthesia

Oxygen was delivered to the canine via face mask, in order to provide mild sedation, reduce aggression and facilitate drug injection [23]. Blood was withdrawn for ABG analysis.

#### General anesthesia

After premedication and mild sedation, the canine’s accurate body weight was measured for precise calculation of drug dosage. General anesthesia was given via intravenous injection of Buprenorphine (0.2 mg/kg) at the sacrococcygeal site. The injection was slowed once symptoms of anesthesia started to appear. The surgical anesthesia symptom was defined as lack of palpebral reflex, muscle relaxation of the jaw tone, and the absence of purposeful movement [22].

The canine was then secured to the operation bed and shaved. Its response was closely monitored as it was becoming unconscious. Observation and interpretation of the canine’s physiologic status were critical to avoid any anesthesia related complications. Body temperature below 37.2 °C should be especially alerted and appropriate measures should be taken to avoid hypothermia.

### Animal model preparation

#### Canine rib fracture model

Under general anesthesia, standard osteotomy was prepared on the canine at the 5th and 6th rib on the left anterior side of the body resulting in oblique fractures. Two 0.5-cm incisions 3 cm apart were made by an electric knife between the 5th and 6th ribs. Through the two incisions, two fracture sites (3 cm apart) were made on the 5th rib, to closely mimic real world trauma injuries. Similarly through the same two incisions, two fracture sites were made on the 6th rib with a total of 4 fracture sites on the canine. The procedure was performed carefully to minimize the damage to the pleura during the operation. The Canine was closely monitored during surgical fixation.

#### Endotracheal intubation

A single-lumen endotracheal tube was placed for proper airway management to avoid any respiratory obstruction. Intubation was gently performed to avoid damage to the larynx. About 5–8 mL of air was injected into the catheter balloon at the distal end, and tubes were secured with tapes. An extended thin tube was used for the relatively long trachea of the canine. Breathing was maintained via a mechanical ventilator.

#### Preoperative measurements

Measurements include: RR (Respiratory Rate, Breath Per Minute), ABG analysis (PO_2_, PCO_2_, and SpCO_2_), Heart Rate (bpm), Body Temperature (℃), Duration of Surgery (Hours), and Total Blood Loss volume (mL).

### Operation procedures

#### Exterior plate fixation

For canines in the Exterior Plate group, the plate was contoured to the appropriate shape and cut into the right size prior to the surgery. Under open surgery, a 5 cm incision was made with an electric knife. The muscles surrounding the broken rib segments were carefully divided. The exposed rib bones were approximated and aligned to the proper positions. The pre-contoured plate was positioned on the 6th rib, and screws of proper size were slowly drilled with 2 screws per fracture side. The same procedure was performed on the 5th rib.

#### Interior plate fixation

##### Incision preparation

**Fig. 2:**
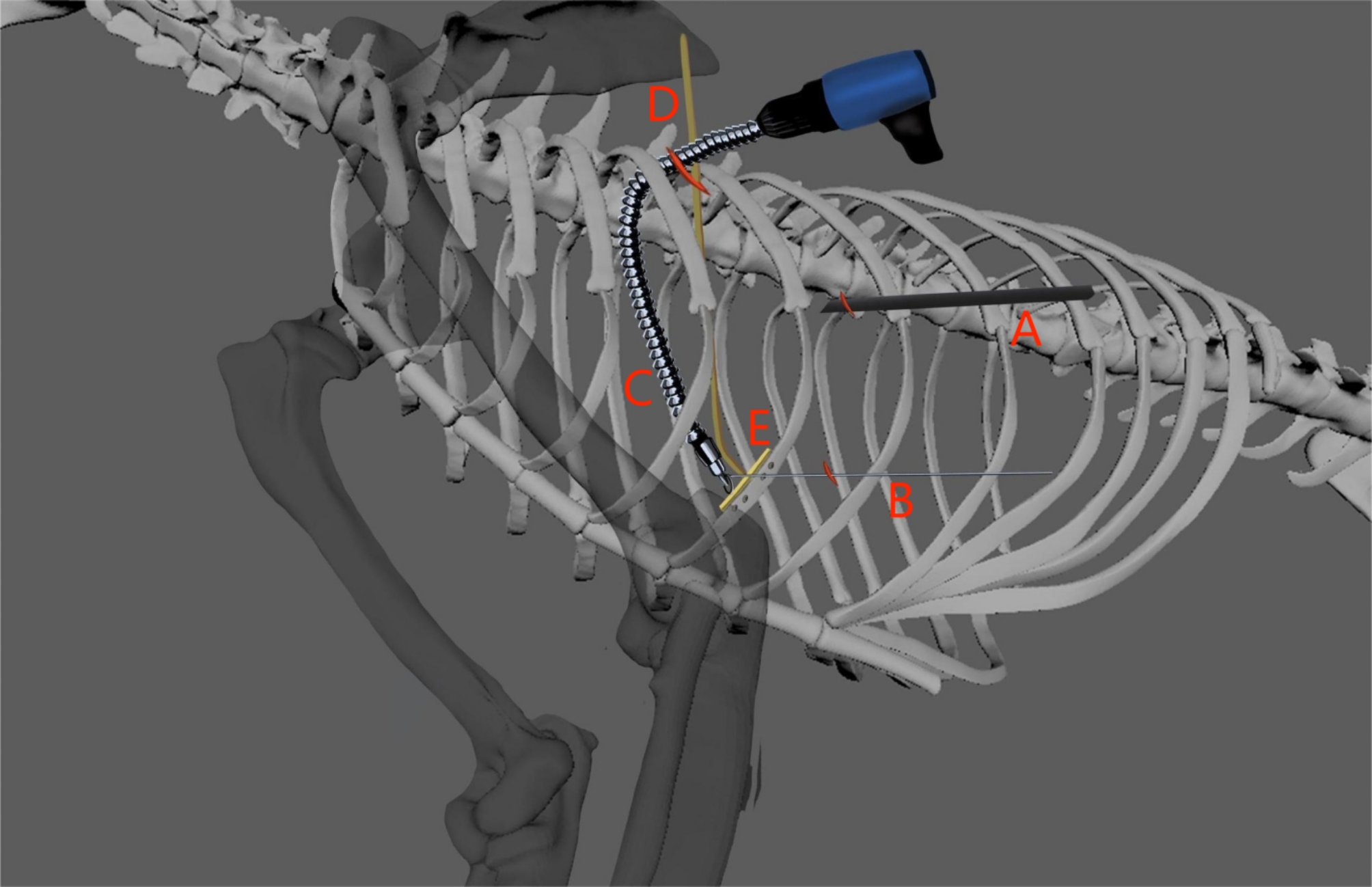
A 3-D Animation of VATS operation setup. **A**. Thoracoscope (for visualization) and CO_2_ inflation; **B**. Guiding Wire with distal Loop (Controlling the tip of the driver); **C**. Flexible Shaft Extension; **D**. Solid Plate Stand (screwed into the metal plate, unscrewed and retrieved after fixation); **E**. Titanium Metal Plate with Threaded Holes (supported and pressed by the plate stand)

There were totally three incisions made to perform the surgery as shown in Fig. 2 marked in red. The 1st incision of 0.5 cm was prepared below the 8th rib along the midaxillary line. A trocar was inserted through the hole and a thoracoscope (shown as A in Fig. 2) was inserted for visualization and inflation of CO_2_ (6 mm Hg pressure) in the thoracic cavity for the collapse of one lung. The 2nd incision of 0.5 cm was prepared below the 7th rib close to the fracture site. A metal wire device with a distal loop (shown as B in Fig. 2) was inserted here to control direction of the flexible shaft extension. The 3rd incision of 2 cm was made along the infrascapular line below the 7th rib on the posterior side for major operations. A circumferential surgical wound retractor was used here. A surgical glove was used to cover the opening to conserve the CO_2_ inflation pressure. A shaft extension with a screwdriver attached to the distal end (shown as C in Fig. 2) and a contoured titanium plate with a solid plate stand (shown as D and E in Fig. 2) were inserted here through the glove cover.

#### Plate description

The titanium plate had seven threaded holes (shown as E in Fig. 2). The solid plate stand (D in Fig. 2) was screwed into the middle threaded hole for firm support during the application process. Other screws were pre-inserted into the threaded holes on the plate (E) to secure their positions so that screws could be easily fastened during the operation.

#### Pre-drilling and fixation

The periosteum on the inward facing side of the 5th rib was carefully divided with an electric knife under thoracoscopic view. The inward facing side of the rib bone was then exposed, and small holes were drilled on the bone at locations guided by the plate holes (E) with the help of flexible shaft extension (C with drill bits attached to the distal end). The threaded plate (E) with pre-inserted screws was then approximated to the desired positions. While the plate was pushed firmly against the interior side of the rib by the rigid plate stand (D), the shaft extension with attached screwdriver (C) was inserted and was guided by the wire loop device (B). The screws were then slowly fastened to avoid thermal damage. After confirming that all screws were securely fastened, the solid plate stand was unscrewed and retrieved. Suction was used to remove any debris from the drilling and screwing. The same procedure was repeated on the 6th rib.

### Postoperative measurements

**Fig. 3:**
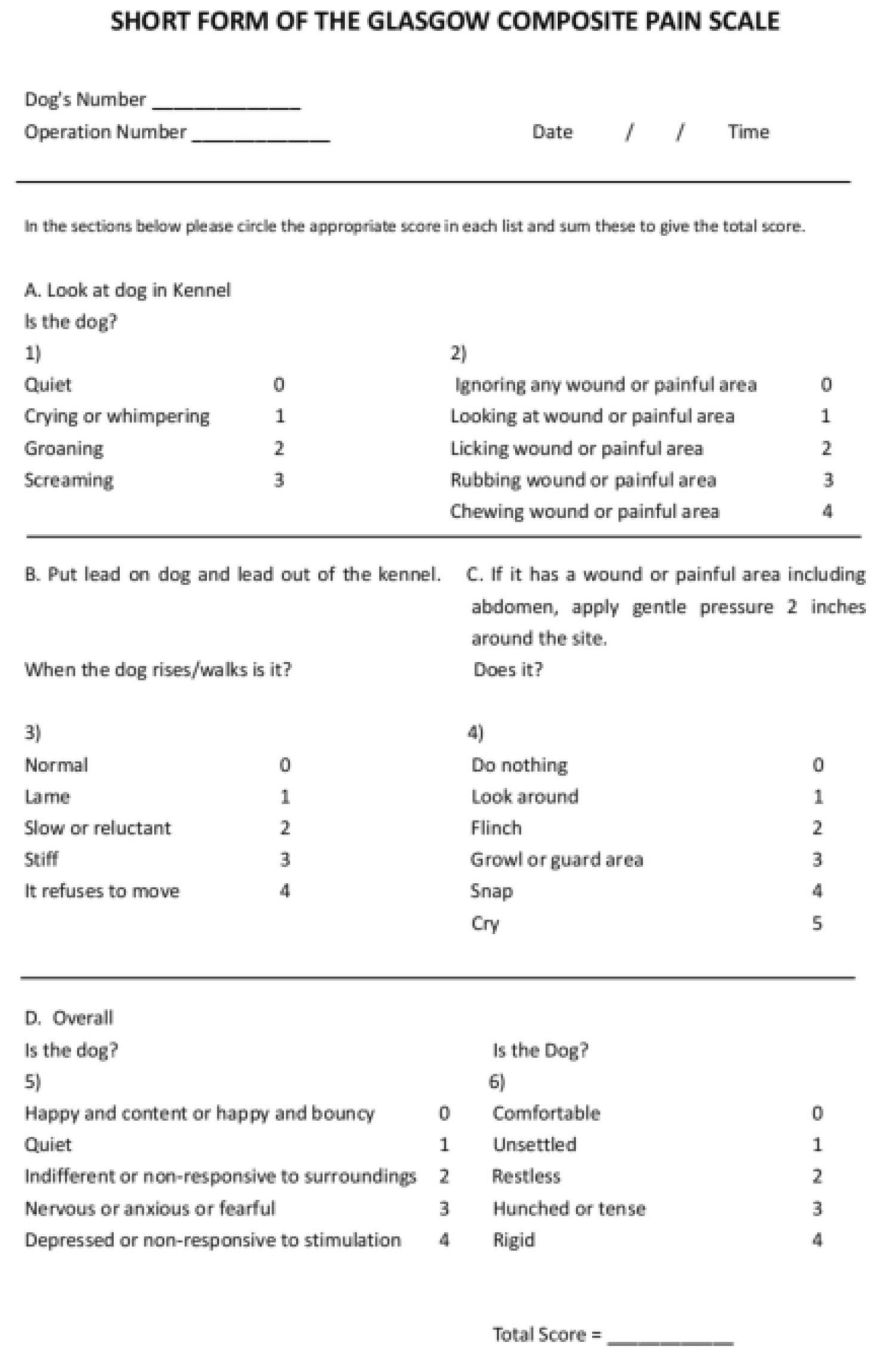
Short Form of the Glasgow Composite Pain Scale, with a score range of 0–24 (0 = less pain, and 24 = more pain)

Postoperative outcomes were recorded 6, 12, 24, and 48 h after surgery. In addition, the Glasgow Composite Measure Pain Scale was assessed within 48 h and up to 14 days after surgery (A blank assessment sheet was shown in Fig. 3) [24]. Other postoperative outcomes were measured to compare the rates of recovery, including: Time to First Food and Water intake, Intake Frequencies, Vomiting and Regurgitation, Time to First Urine and Stool, Excretion Frequencies, Time to First Standing and Gentle Ambulation, and Mobilization Frequencies up to 14 day after operation. The outcomes were measured and recorded by assessors blinded to the groups. Long term recovery outcomes were not recorded due to limited resources.

### Statistical analysis

The IBM SPSS Statistics 26 software for Windows (SPSS Inc., Chicago, IL, USA) was used for data processing and analysis. Independent-Samples T Test was used to compare functions and outcomes. Data was given as Mean ± S.D. or Median (Interquartile Range) with 95% confidence interval. All testing was two tailed, and a *P* value less than 0.05 was considered to be statistically significant.

## Results

The surgical fixation was performed successfully on all 12 canines without major complication or respiratory aggression. Respiratory and cardiovascular functions were closely monitored and recorded throughout the operation to reflect the physiologic status of the canines under anesthesia. The difference in preoperative measurements on respiratory functions, cardiovascular functions and body temperature were not statistically different between the two groups, with *P* values greater than 0.05, as shown in Table 1.

Several postoperative outcomes were measured and recorded to compare the rate of recovery from the two rib stabilization methods. The difference in postoperative respiratory rates were not statistically significant between the two groups 6 and 12 h after the surgery (with *P* values greater than 0.05) as shown in Table 1. The respiratory rates of the canines in the interior group became slower than the exterior group 24 and 48 h after surgery (*P* values of 0.018 and 0.001 respectively).

The ABG analysis results (PCO_2_, PO_2_, SpO_2_) and Heart Rates of the two groups didn’t significantly differentiate 6 h, 12 h, or 24 h after surgery (with *P* values greater than 0.05), except for the PO_2_ values 24 h post operation (89.83 mmHg in EP and 92.00 mmHg in IG with *P* value of 0.021), as shown in Table 1. The difference in PCO_2_, PO_2_, SpO_2_, and HR were statistically significant between the two groups 48 h after the surgery (*P* values less than 0.05). The interior group canines had lower PCO_2_ and HR, higher PO_2_ and SpO_2_ values than canines in the exterior group in the 48^th^ hour. The statistically significant difference in respiratory and cardiovascular functions between the two groups 48 h after surgery showed that the Interior Group canines had better recovery outcomes in terms of lung and heart functions.

The difference in body temperature between the two groups was not statistically significant between the two groups with *P* values greater than 0.05.

### Preoperative and postoperative measurements

**Table 1:** Preoperative and Postoperative Measurements. Data was given as Mean ± S.D. with 95% confidence interval. *P* value was calculated using Independent-Samples T Test, and a *P* value less than 0.05 was considered to be statistically significant. Pre, Pre-anesthesia Measurements, RR, Respiratory Rate. PCO_2_, Partial pressure of carbon dioxide. PO_2_, Partial pressure of oxygen. SpO2, Saturation of Pulse Oxygen. HR, Heart Rate. Temp, Body Temperature. DS, Duration of Surgery. TBL, Total Blood Loss volume

Factors		Exterior Group	Interior Group	*P* value
**RR, Breath/Min**	**Pre**	18.50 ± 1.64	18.00 ± 1.41	0.636
	**6 h**	31.50 ± 1.64	30.50 ± 1.05	0.237
	**12 h**	28.67 ± 2.25	26.00 ± 2.00	0.055
	**24 h**	26.50 ± 1.64	24.00 ± 1.41	**0.018**
	**48 h**	24.83 ± 1.47	21.33 ± 1.21	**0.001**
**PCO**_**2**_, **mmHg**	**Pre**	37.00 ± 1.67	36.33 ± 1.86	0.175
	**6 h**	40.17 ± 1.17	39.83 ± 1.17	0.632
	**12 h**	40.00 ± 1.67	40.33 ± 1.86	0.751
	**24 h**	39.50 ± 2.81	39.67 ± 1.97	0.908
	**48 h**	39.50 ± 1.05	36.83 ± 1.47	**0.005**
**PO**_**2**_, **mmHg**	**Pre**	89.33 ± 3.27	89.67 ± 2.66	0.793
	**6 h**	89.50 ± 2.59	90.50 ± 2.07	0.477
	**12 h**	89.33 ± 2.25	91.17 ± 1.72	0.144
	**24 h**	89.83 ± 1.72	92.00 ± 0.89	**0.021**
	**48 h**	89.50 ± 1.38	92.00 ± 0.83	**0.004**
**SpO**_**2**_, **%**	**Pre**	96.17 ± 0.41	96.50 ± 1.05	0.363
	**6 h**	95.17 ± 0.41	95.83 ± 0.75	0.086
	**12 h**	94.17 ± 1.60	95.00 ± 1.41	0.362
	**24 h**	95.00 ± 2.28	96.50 ± 1.38	0.198
	**48 h**	95.67 ± 1.03	97.33 ± 0.82	**0.011**
** h,bpm**	**Pre**	89.33 ± 4.80	85.00 ± 3.79	0.184
	**6 h**	98.33 ± 4.80	96.50 ± 1.22	0.386
	**12 h**	95.50 ± 5.75	92.00 ± 3.79	0.242
	**24 h**	93.33 ± 3.56	90.83 ± 4.79	0.329
	**48 h**	93.67 ± 3.14	89.67 ± 1.63	**0.020**
**Temp, °**	**Pre**	38.17 ± 0.52	38.38 ± 0.70	0.492
	**6 h**	38.78 ± 0.60	38.28 ± 0.23	0.085
	**12 h**	38.67 ± 0.52	38.68 ± 0.70	0.963
	**24 h**	38.90 ± 0.11	38.88 ± 0.52	0.941
	**48 h**	38.83 ± 0.41	38.55 ± 0.47	0.292

The Exterior Group operation took shorter time than the Interior Group (45.83 ± 2.04 min in EG and 68.50 ± 2.43 min in IG respectively, with P value less than 0.001), which indicated that the Exterior fixation was quicker to perform than the interior minimally invasive procedure. However, the Exterior Group had higher total blood loss volumes (50.00 ± 3.22 mL in EG and 33.33 ± 1.86 mL in IG with P value less than 0.001) than the Interior Group.

The pain scale was evaluated and recorded by blinded assessors 1, 3, 6, 12, 24, and 48 h after the surgery, and once every day on Day 3 through Day 14, as shown in Table 2. The median pain scores of the canines in the Exterior Group (11.50 within 48 h, 9.00 within 7 days, and 6.50 within 14 days) were higher than scores in the Interior Group (8.50 within 48 h, 7.00 within 7 days and 4.00 within 14 days), with *P* values of 0.002, 0.001 and less than 0.001. The difference in pain scores were statistically significant. Lower pain scores in the Interior Group showed that the canines suffered less pain.

### Postoperative pain assessment

**Table 2:** Postoperative Pain Assessment. Data was given as Median (Interquartile Range) with 95% confidence interval. *P* value was calculated using Independent-Samples T Test, and a *P* value less than 0.05 was considered to be statistically significant

Factors	Exterior Group	Interior Group	*P* value
**Pain Scale within 48 h (0–24)**	11.5 (10.75 -12.00)	8.50 (8.00–9.00)	**0.002**
**Pain Scale within 7 Days (0–24)**	9.00 (8.75–9.00)	7.00 (6.00–7.00)	**0.001**
**Pain Scale within 14 Days (0–24)**	6.50 (6.00–7.00)	4.00 (4.00–4.25)	**< 0.001**

Gastrointestinal Mobility was evaluated to compare the recovery outcomes of the two groups, as shown in Table 3. It took canines in the Exterior Group longer time to start food intake (12.00 h in EG, 7.83 in IG with a *P* value of 0.001). The canines in EG also had lower Food Intake Frequencies within 7 days and 14 days with *P* values of 0.005 and less than 0.001. The difference in Time to First Water Intake was not statistically significant in the two groups, and the interior group had higher Water Intake Frequencies in the three time intervals with all three *P* values less than 0.001. The higher frequency of food and water intake in the Interior Group showed quicker appetite recoveries. However, the difference in the time to first urine and stool excretion and the excretion frequencies was not statistically significant, with *P* values greater than 0.05.

### Postoperative gastrointestinal mobility

**Table 3:** Postoperative Gastrointestinal Mobility. Data was given as Mean ± S.D. with 95% confidence interval. P value was calculated using Independent-Samples T Test, and a P value less than 0.05 was considered to be statistically significant

Postoperative Gastrointestinal Mobility				
Factors		Exterior Group	Interior Group	*P* value
**Time to First Food Intake (Hrs)**		12.00 ± 1.41	7.83 ± 0.75	**0.001**
**Food Intake Frequency**	**48 h**	1.83 ± 0.75	2.83 ± 0.75	0.111
	**7 Days**	13.50 ± 1.05	15.83 ± 1.17	**0.005**
	**14 Days**	26.50 ± 1.87	35.00 ± 1.41	**< 0.001**
**Time to First Water Intake (Hrs)**		2.83 ± 0.75	2.50 ± 1.05	0.363
**Water Intake Frequency**	**48 h**	9.50 ± 1.05	14.83 ± 1.17	**< 0.001**
	**7 Days**	28.17 ± 2.32	37.67 ± 2.16	**< 0.001**
	**14 Days**	62.00 ± 2.37	84.67 ± 3.44	**< 0.001**
**Vomiting and Regurgitation within 48 h**		2.00 ± 1.41	1.50 ± 1.05	0.542
**Time to First Urine (Hrs)**		4.50 ± 1.05	4.50 ± 1.05	1
**Urine Frequency**	**48 h**	2.67 ± 0.82	2.83 ± 1.17	0.822
	**7 Days**	9.83 ± 1.47	10.83 ± 1.47	0.253
	**14 Days**	34.00 ± 2.61	35.17 ± 1.17	0.272
**Time to First Stool (Hrs)**		5.50 ± 1.05	5.67 ± 1.21	0.809
**Stool Frequency**	**48 h**	1.17 ± 0.75	1.50 ± 1.05	0.638
	**7 Days**	9.17 ± 1.17	10.17 ± 1.72	0.377
	14 Days	19.83 ± 1.94	20.33 ± 1.75	0.611

It took the EG canines longer time to fully stand without assistance (15.83 h in EG, 12.00 h in IG, with a *P* value of 0.016), and longer time to start gently walking around (20.50 h in EG, 14.5 h in IG, with a *P* value of 0.002), as shown in Table 4. The canines in the Exterior Group had lower mobilization frequencies within 48 h, 7 days and 14 days after the surgery, with *P* values of 0.013, 0.005, and less than 0.001. Shorter time to fully stand and gentle ambulation, together with higher mobilization frequencies showed that the canines in interior group recovered quicker and better to normal physical function.

**Table 4:** Postoperative Physical Mobility. Data was given as Mean ± S.D. with 95% confidence interval. *P* value was calculated using Independent-Samples T Test, and a *P* value less than 0.05 was considered to be statistically significant

Postoperative Physical Mobility			
Factors	Exterior Group	Interior Group	*P value*
**Time to Standing without Assistance (Hrs)**	15.83 ± 1.47	12.00 ± 1.79	**0.016**
**Time to First Gentle Ambulation (Hrs)**	20.50 ± 1.05	14.50 ± 1.87	**0.002**
**Mobilization Frequency within 48 h**	5.50 ± 1.05	9.00 ± 1.41	**0.013**
**Mobilization Frequency within 7 Days**	35.33 ± 2.16	42.17 ± 1.72	**0.005**
**Mobilization Frequency within 14 Days**	85.33 ± 1.86	104.5 ± 3.78	**< 0.001**

## Discussion

The difference in preoperative measurements of the canines were not statistically significant in the two groups, indicating that any changes in postoperative measurements were effects of the two operation techniques. No serious operational complications were observed during and after the surgeries, which showed that both procedures were safe to perform. The exterior fixation took less operation time due to open surgery approach. The metal plate was applied under direct vision without the application of complicate tools and devices. However, the interior fixation led to less blood loss as results of small incisions and less damage to tissues and blood vessels surrounding the rib.

The postoperative results (RR, PCO_2_, PO_2_, SpO_2_, and HR) of the canines in the two groups didn’t significantly differentiate within 12 h post operation. Although canines tend to recover from trauma quickly, their physiological parameters and physical movements were slow in returning to normal levels in both groups in the first 24 h of operation, due to the traumatic event, general anesthesia and surgical operations. However, after the initial post-surgery period, the canines in the Interior Group started to show more signs of recovery comparing to canines in the Exterior Group.The RR and PO2 values of canines in the interior group showed better recovery starting from the 24th^th^ hour and the interior group PCO_2_, SpO_2_, and HR values started to show better results since the 48^th^ hour after surgery, indicating quicker respiratory and cardiovascular function recoveries than the exterior group canines.

The Body Temperature results didn’t significantly differentiate between the two groups before or after the operation. The canines in the Interior Group had lower pain scores, because the minimally invasive procedure didn’t require the splints of muscles, resulting in less neurovascular damages and less pain.

It took the EG canines longer time to start eating, and their food and water intake frequencies were lower than in the Interior Group. The delayed return to normal appetite for the EG canines showed that the interior fixation method led to quicker recovery to normal gastrointestinal mobility. However, the difference in vomiting, urine and stool data was not statistically significant between the two groups.

The results also showed that the IG canines had quicker recovery to normal physical function. With less injuries from the interior fixation, it took the IG canines less time to start standing and walking, and they were more willing to move around than the EG canines.

Flail chest as a result of multiple rib fractures is very common after blunt trauma injury, and it is often associated with severe morbidity and high mortality rates. Current treatment options remain controversial.

Traditional management of flail chest recommends supportive and non-operative treatment, such as active anesthesia with mechanical ventilation support. Conservative management can sometimes lead to reliance on pain medicine and mechanical ventilation, and the long-term ICU stay is costly. The healing and re-union of the fractured ribs are poor and slow. Chest deformity is common after the injury, and the rate of pulmonary infection is high [25].

Several studies have suggested that timely surgical fixation could lead to quicker release from ICU and that better post-operative outcomes could be achieved with the proper fixation method selected [11, 26]. Most importantly, the rate of pulmonary complications is lower comparing to that in non-operative treatments.

A variety of surgical fixation methods have been designed and tested in recent years. Thus far, there has not been a universally accepted optimal fixation option. Most of the commercially available products have both advantages and deficiencies. Designs and the testing of new fixation devices are clinically necessary in order to achieve better patient outcomes.

Metal prostheses provide adequate holding strength after placement. However, they could be contraindications to future MRI/CT scanning. These metal prostheses could also lead to stress-shielding, where the natural bone growth is interrupted by the strength of metals [19]. Loosening of the prosthesis is also common after continual forceful movement by breathing, coughing or sneezing. A follow-up removal surgery is sometimes necessary after initial implantation, which is costly and painful [20, 21].

Even with the above-mentioned setbacks, many studies on surgical fixation have focused on metal devices. One of the popular devices is the set of straight or pre-contoured titanium plates of various sizes. These plates are contoured and cut into the proper size and applied on the outward facing side of the rib. The application of these plates requires careful division of the muscles surrounding the rib for proper positioning and stabilization. However, the division process can damage intercostal nerves and blood vessels around the rib bones, causing bleeding and chronic pain after implantation. Tissue migration onto the metal plates can also be a challenge to potential future removal.

The novel interior fixation method is designed to minimize tissue division by application of plates on the inward facing side of the ribs. Only a thin layer of periosteum needs to be divided to expose the bone. Surrounding structures (Intercostal muscles, nerves and blood vessels) were mostly preserved during the fixation, minimizing adverse tissue reactions, hypersensitivity, or chronic pain.

This interior application could be technically difficult due to limited operation space and challenging angles. In this study, a few surgical tools were designed to overcome the challenges. A flexible soft shaft extension can be bent, twisted, and rotated into various angles with the help of a guiding wire loop. Previously inserted screws on the threaded metal plates can secure the positions while the force is being applied. The solid plate stand is also screwed into the plate to firmly support the metal plate by pressing against the bone during fixation. The stand can be easily unscrewed from the plate and retracted after the fixation.

The experiment results in this study demonstrated that the interior fixation method was safe to use, and it led to quicker and better recoveries after surgery, in terms of respiratory and cardiovascular functions, pain assessment, gastrointestinal and physical function. The experiment results could be clinically meaningful, because further preclinical and clinical studies could focus more on the interior fixation over the exterior fixation. Most of the current surgical methods were centered on the design and testing of various devices to stabilize the ribs on the outward facing side, requiring the splints of muscles and nerves on the rib, resulting in pain and slow recovery. This animal experiment results demonstrated that it was safe and effective to stabilize the broken ribs from the inward facing side, with less pain and quicker recovery to normal gastrointestinal and physical activities.

Although all operations were successful during the experiment, a few deficiencies still existed. The body line of canine is parallel to the ground, while it is perpendicular to the ground in human. The difference in body line angle means that gravity force on the interior attachment of metal prosthesis in canines is supported by the ribs themselves; while in human, the gravity force on metal plate does not have the same level of support as in dogs. Possible complication might exist when applying the fixation method in clinical use, so that stronger attachment would be needed if applied to human. The interior fixation method took longer time than the open surgery exterior fixation. Due to limited resources, all canines were not kept for long-term follow up. Straight and transverse fractures were made during the osteotomy, but practically, oblique fractures occur more often in blunt trauma. Also, in the real world of emergency trauma management, the timing and urgency of the situation sometimes do not allow minimum invasive operation to be performed. Only patients in stable living condition are suitable for VATS fixation under one-lung ventilation [27]. Further investigations and improvements are necessary before clinical trials. This study provides alternative solutions to surgical fixation on rib fracture.

## Conclusions

The investigative interior metal plate fixation method was safe and effective in rib stabilization on a canine model, comparing to the open surgery exterior fixation method. The Interior fixation method was minimally invasive, causing less damage to tissues and nerves surrounding the ribs, leading to better postoperative outcomes.

### Electronic supplementary material

Below is the link to the electronic supplementary material.

Supplementary Material 1

## Data Availability

The datasets used and/or analyzed during the current study are available from the corresponding author on reasonable request.

## List of abbreviations

VATS: Video assisted Thoracoscopic Surgery
ARDS: Acute Respiratory Distress Syndrome
ICU: Intensive Care Unit
CT: Computerized Tomography
MRI: Magnetic Resonance Imaging
RR: Respiratory Rate
PCO_2_: Partial pressure of carbon dioxide
PO_2_: Partial pressure of oxygen
SpO2: Saturation of Pulse Oxygen
HR: Heart Rate
Temp: Body Temperature
DS: Duration of Surgery
TBL: Total Blood Loss volume
EG: Exterior Group
IG: Interior Group
